# IS THE NEW PROCORE 20G DOUBLE FORWARD-BEVEL NEEDLE CAPABLE TO OBTAIN BETTER HISTOLOGICAL SAMPLES BY ENDOSCOPIC ULTRASOUND FOR DIAGNOSING SOLID PANCREATIC LESIONS?

**DOI:** 10.1590/0102-672020200004e1554

**Published:** 2021-01-25

**Authors:** José Celso ARDENGH, Vitor Ottoboni BRUNALDI, Mariângela Ottoboni BRUNALDI, Alberto Facuri GASPAR, Jorge Resende LOPES-JÚNIOR, Ajith Kumar SANKARANKUTTY, Rafael KEMP, José Sebastião dos SANTOS

**Affiliations:** 1 Division of Gastrointestinal Surgery (Endoscopy Unit), Department of Surgery and Anatomy, Ribeirão Preto Medical School, University of São Paulo, São Paulo, Brazil; 2 Hospital das Clínicas, Ribeirão Preto Medical School, University of São Paulo, São Paulo, Brazil; 3 Department of Pathology and Forensic Medicine, Ribeirão Preto Medical School, University of São Paulo, São Paulo, Brazil

**Keywords:** Diagnosis, Endoscopic Ultrasound, Fine-Needle aspiration biopsy, Fine-needle biopsy, Pancreatic cancer, Diagnóstico, Endossonografia, Biópsia, Neoplasias Pancreáticas, Patologia

## Abstract

**Background:**

It is important to obtain representative histological samples of solid biliopancreatic lesions without a clear indication for resection. The role of new needles in such task is yet to be determined.

**Aim:**

To compare performance assessment between 20G double fine needle biopsy (FNB) and conventional 22G fine needle aspiration (FNA) needles for endoscopic ultrasound (EUS)-guided biopsy.

**Methods:**

This prospective study examined 20 patients who underwent the random puncture of solid pancreatic lesions with both needles and the analysis of tissue samples by a single pathologist.

**Results:**

The ProCore 20G FNB needle provided more adequate tissue samples (16 vs. 9, p=0.039) with better cellularity quantitative scores (11 vs. 5, p=0.002) and larger diameter of the histological sample (1.51±1.3 mm vs. 0.94±0.55 mm, p=0.032) than the 22G needle. The technical success, puncture difficulty, and sample bleeding were similar between groups. The sensitivity, specificity, and diagnostic accuracy were 88.9%, 100%, and 90% and 77.8%, 100%, and 78.9% for the 20G and 22G needles, respectively.

**Conclusions:**

The samples obtained with the ProCore 20G FNB showed better histological parameters; although there was no difference in the diagnostic performance between the two needles, these findings may improve pathologist performance.

## Graphical abstract

[Fig f1a]

**Figure f1a:**
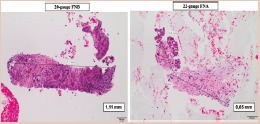
Fine needle biopsy (FNB) histological samples by endoscopic ultrasound

[Fig f1as]

**Figure f1as:**
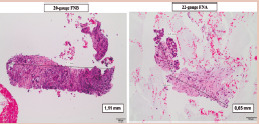
Amostra histológica de biópsia por agulha fina (FNB) por ultrassonografia endoscópica

## RESUMO

**Racional:**

As lesões sólidas pancreáticas não ressecáveis cirurgicamente demandam boa amostragem tecidual para definição histológica e condução oncológica** .** O papel das novas agulhas de ecopunção no aprimoramento diagnóstico ainda necessita elucidação.

**Objetivo:**

Comparar as biópsias guiadas por ecoendoscoopia com a nova agulha 20G de bisel frontal duplo (FNB) com a agulha de aspiração fina 22G convencional.

**Métodos:**

Este estudo prospectivo avaliou 20 pacientes submetidos à punção de lesões pancreáticas sólidas com ambas agulhas e envolveu análise de amostras teciduais por um único patologista.

**Resultados:**

A agulha FNB 20G forneceu amostras de tecido mais adequadas (16 vs. 9, p=0,039) com melhores escores quantitativos de celularidade (11 vs. 5, p=0,002) e maior diâmetro máximo da amostra histológica (1,51±1,3 mm vs. 0,94±0,55 mm, p=0,032) que a agulha 22G. O sucesso técnico, dificuldade de punção e sangramento da amostra foram semelhantes entre os grupos. A sensibilidade, especificidade e acurácia diagnóstica foram 88,9%, 100% e 90% e 77,8%, 100% e 78,9% para as agulhas 20G e 22G, respectivamente.

**Conclusão:**

As amostras obtidas com a FNB 20G apresentaram melhores parâmetros histológicos, embora não tenha havido diferença no desempenho diagnóstico entre as duas agulhas.

## INTRODUCTION

Obtaining cells by endoscopic ultrasound-guided fine-needle aspiration (EUS-FNA) is crucial for the histological diagnosis of solid pancreatic lesions^25^ with no indication for surgical resection^8^ ^,^ ^28^. The sensitivity, specificity, positive predictive value (PPV), negative predictive value (NPV), and accuracy of detecting pancreatic carcinoma (PC) are 79-98%, 71-100%, 96-100%, 33-85%, and 82-98%, respectively^,17-19,21,23,24,26,29,30^, while the false negative and false positive rates are 12-14%^1^ ^,^ ^31^ and 0-5%^1^ ^,^ ^14^ ^,^ ^16^ ^,^ ^31^, respectively. Therefore, efforts to improve the collection of tissue samples for histological diagnosis and additional molecular tests can increase the accuracy of the biopsy obtained by EUS and positively influence daily clinical practice, research, and treatment. Considering the FNA methodology, device positioning, analysis by a specialized pathologist, needle gauge, lesion characteristics and location, and the technical variables for obtaining and studying solid pancreatic lesion samples, can interfere with the diagnostic accuracy of EUS-FNA^10^ ^,^ ^25^. In this context, new needle biopsy (fine needle biopsy [FNB) devices that can collect tissue cores instead of just cytological samples can theoretically improve diagnostic accuracy without affecting the technique, costs, and occurrence of adverse events. 

The diagnostic performance of EUS-FNA using the samples obtained with these needles was recently evaluated^7^ ^,^ ^9^, but different FNB types are available that have not yet been compared^13^. The newly designed 20G double forward-bevel FNB needle (ProCore) is one of these new devices that aims to improve the diagnostic capacity of the histological samples obtained by EUS-FNA.

Recent meta-analyses that assessed the diagnostic capacity of the histological samples obtained did not include studies with the new 20G double forward-bevel FNB needle (ProCore)^12^ ^,^ ^13^. 

Therefore, this study aimed to compare the performance of the 22G needle and the new 20G needle (ProCore) in obtaining the tissue sample and in the diagnosis of solid pancreatic tumors.

## METHODS

### Study design

This prospective randomized study was conducted at Hospital das Clínicas, Ribeirão Preto Medical School, University of São Paulo, Ribeirão Preto, São Paulo, Brazil), a public and tertiary teaching hospital, upon receiving approval from its research ethics committee (process number 6971-2018).

### Eligibility criteria

Adult patients (>17 years old) with solid pancreatic lesions referred for diagnosis by EUS-FNA, excluding those with cystic lesions, clinical instability, intractable coagulopathy (international normalized ratio <1.5 and/or thrombocytopenia <50,000), previous pancreatic biopsy, and severe ascites as well as those who refused to sign the informed consent form.

### Randomization and allocation

The randomization list was created using a system based on a computer program in the proportion of 1:1^31^. The randomization list and allocation envelopes were defined before the first inclusion. Based on the list, a researcher uninvolved with the study prepared sealed sequential opaque envelopes that were opened upon confirmation of the patient’s eligibility during the EUS assessment.

All patients were submitted to four needle passes (22G and 20G) with 10 back-and-forth movements during each pass and were allocated into two groups based on the needle type used first. Patients in group A underwent two punctures with a 22G FNA needle followed by two punctures with a 20G FNB needle, while those in group B underwent two punctures with a 20G needle followed by two punctures with a 22G needle. The pathologist was aware of the type of needle used for the collection but was unaware of the allocation group.

### Procedures

All patients underwent EUS under deep sedation with propofol. The standard biliopancreatic assessment was initially performed using a linear echoendoscope (Fujinon 530-UT or Pentax EG-3870UTK). Eligibility was confirmed after the collection of demographic and clinical data, results of previous imaging tests and EUS findings were reviewed, a researcher involved with the study opened the allocation envelope, and the EUS-guided punctures were performed. A 22G FNA needle (Expect; Boston Scientific, Marlborough, MA, USA) and a 20G FNB needle (Procore 20G, Cook Medical, Bloomington, IN, USA) were used.

The needle was passed through the lesion and an image was captured to confirm its correct positioning. Next, the stylet was completely removed and suction was performed with a 10-cc syringe. The ventilation technique was also used to improve tissue sample collection^35^. After 10 back-and-forth movements, the needle was retracted and washed with saline to extract the sample collected in a 10% formalin vial to the block. As each vial represented a passage of the needle, each patient had four vials identified with needle type. An experienced endoscopist performed all procedures (JCA) and graded the puncture difficulty (1:none; 2:easy; 3:moderate; 4:difficult) and reported the macroscopic impression of the amount of tissue collected (1:little; 2:regular; 3:increased).

After the puncture, the endoscopist assessed the gastrointestinal tract for immediate adverse events (AEs). The patients were sent to the recovery room, observed for about 2 h after the procedure, and subsequently discharged if no AE was detected and provided a phone number of the service for contact if needed. An outpatient visit was scheduled about one month after the procedure to examine the result and assess any late AEs. Early adverse events were defined as those starting within 48 h after the procedure and late events as those starting within 48 h to one month after the procedure.

### Histological assessment

The samples were previously fixed in 10% formalin for 6-24 h. Fragments larger than 1 mm were processed with a standard paraffin block. Samples smaller than 1 mm were centrifuged (1.500 rpm) for 10 min. The tissue sediment was collected in Eppendorf tubes containing 1.5 ml of 3% agarose and then centrifuged again. The cell block immersed in agarose was cooled to 0.7°C. After solidifying, the block was sectioned, placed in cassettes, and sent for standard histological assessment with paraffin inclusion^5^.

A specialized pathologist (MOB) assessed all samples. The cellularity and bleeding were assessed using validated objective scales. The cellularity was classified from 1 to 4 as follows: 1,<50 cells; 2, 50-100 cells; 3, 100-200 cells; 4, >200 diagnostic cells. Scores 3 and 4 were considered adequate for histological assessment^27^. The bleeding was graded as mild (scarce hemorrhagic cells), moderate (frequent hemorrhagic cells), or intense (frequent hemorrhagic cells and clots that impair the histological assessment). The cell groups were measured using the cellSens Micro Imaging Software (Olympus America Inc, Center Valley, PA, USA). The size of the largest histological nucleus was annotated for each pass and each needle for later comparison.

### Gold standard for comparison

In cases indicated for surgical resection, the histopathology of the resected sample was the diagnostic gold standard. For patients who did not undergo surgery, the final diagnosis of malignancy was provided by disease progression considering clinical deterioration, death, and/or images consistent with metastatic lesions after six months of follow-up. Similarly, the negative gold standard in cases of a negative histopathological diagnosis was a consistent clinical result and images after six months of follow-up. In addition to adenocarcinoma, pancreatic neuroendocrine tumors and mesenchymal neoplasms were also considered positive for malignancy.

### Endpoints

The primary endpoint was the proportion of suitable samples obtained with each needle. The secondary endpoints included the endoscopist’s impression of the tissue amount and puncture difficulty during EUS, larger cell group size, classification of bleeding in the histological assessment, and diagnostic efficacy of each needle in terms of sensitivity, specificity, positive and negative predictive value, and accuracy.

### Statistical analysis

Continuous variables are expressed as medians and means, while categorical data are shown as frequencies and proportions. Fisher’s exact test was used to assess the correlation between the diagnostic accuracy of each technique and McNemar’s test to compare the proportion of suitable samples. The Wilcoxon test was used to analyze cellularity, bleeding, and histology. Cohen’s kappa statistic was used to measure agreement between needles. P values ≤0.05 were considered significant with a 95% confidence interval. The sensitivity, specificity, positive and negative predictive values, and accuracy were calculated using standard definitions. The paired proportion test was used to compare the sensitivities and specificities of the needles.

## RESULTS

A total of 64 patients were referred for EUS between December 2017 and April 2018. Of these, 20 met the eligibility criteria and were included in the study. The demographic, clinical, and imaging data are shown in Table 1. The EUS showed that the pancreatic lesions were a mean 37±19 mm in the largest diameters and 11 were Doppler-negative masses. Qualitative elastography showed four soft tumors, seven intermediate tumors, and nine hard masses. Two patients were diagnosed with metastatic disease after the EUS assessment. Regarding the subjective classification of the number of collected specimens and the technical difficulty during the punctures, the 20G FNB needle provided a better impression of “increased material” compared to the 22G FNA needle (p<0.001) with a similar difficulty profile (Table 2).

**FIGURE 1:**
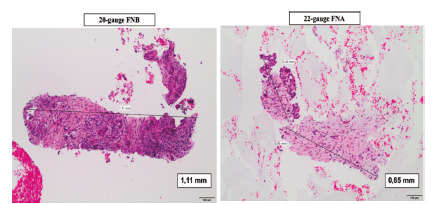
Histological specimens from the same patient with the 20-gauge FNB needle (left) vs. the 22-gauge FNA needle (right, H&Ex100)

The histopathological assessment showed that the mean diameter of the largest sample was 1.51 ± 1.3 mm and 0.94 ± 0.55 mm for the 20G FNB and 22G FNA needles, respectively (p=0.032). The FNB needle allowed the collection of larger samples (6.3 mm wide). Figure 1 shows a histological comparison of the largest sample for the same patient with the different needles.

The number of cases with >200 diagnostic cells was higher for the 20G FNB needle than the 22G FNA needle (p=0.002). Consequently, the FNB provided more suitable samples than the FNA (16 vs. 9, p=0.039). There were no differences in terms of bleeding based on the worst score for each needle (p=0.655). Table 3 summarizes the histopathological findings.

**TABLE 1:** Demographic, clinical, and imaging data of 20 patients who underwent pancreatic solid lesion puncture using endosonography

Age (years)	64.7±12.5
Gender	
M	9 (45%)
F	11 (55%)
Clinical history of cancer	
Colorectal	2 (10%)
Kidney	1 (5%)
Uterus	1 (5%)
Symptoms	
Abdominal pain	14 (70%)
Weight loss	12 (60%)
Lower back pain	9 (45%)
Jaundice	8 (40%)
Nausea/vomiting	5 (25%)
Diarrhea	2 (10%)
Imaging findings (CT/MRI)	
Hypovascular	12 (60%)
Hypervascular Isovascular	6 (30%) 2 (10%)
Contours	
Smooth	11 (55%)
Irregular	9 (45%)
Location	
Head	12 (60%)
Body	6 (30%)
Tail	2 (10%)
Largest diameter (cm)	3.81 ± 2.18
Ascending MPD dilation	12 (60%)
Distal atrophy	8 (40%)

cm=centimeter; CT=computed tomography; F=female; M=male; MPD=main pancreatic duct; MRI=magnetic resonance

**TABLE 2:** Endosonographer’s impression of the amount of procured tissue and technical difficulty during puncture according to the needle employed

		Needle				p*
		22G FNA		20G FNB		
		n	%	n	%	
Procured specimen	Low	3	15	0	0	<0.001
	Moderate	15	75	2	10	
	Augmented	2	10	18	90	
Technical difficulty	None	15	75	11	55	0.323
	Easy	3	15	6	30	
	Intermediate	1	5	1		
	Hard	1	5	2	10	

FNA=fine needle aspiration; FNB=fine needle biopsy

**TABLE 3:** Comparison of needles in terms of best cellularity and worst bleeding

	Needle				p*
	22G FNA		20G FNB		
Cellularity	n	%	n	%	
<50 diagnostic cells	6	30	0	0	0.002
50-100 diagnostic cells	5	25	4	20	
100-200 diagnostic cells	4	20	5	25	
>200 diagnostic cells	5	25	11	55	
Bleeding	n	%	n	%	p*
Low	6	30	6	30	0.655
Moderate	14	70	13	65	
Severe	0	0	1	5	
					

Only one sample had no cells for diagnosis using the FNA needle, which was considered technical failure. A total of 14 and 16 cases had a positive diagnosis of malignancy with the 22G FNA and 20G FNB needles, respectively (p=0.50). Later, 18 cases were confirmed positive during follow-up or surgery, indicating a sensitivity of 77.8% and 88.9% for the 22G FNA and 20G FNB needles, respectively. On the paired proportions test, there were no differences in needle sensitivity (p=0.36). There were no false positives for malignancy. The two true negative cases were diagnosed with autoimmune pancreatitis (n=1) and serous microcystic adenoma (n=1). The accuracy rates were 78.9% and 90% for the 22G FNA and 20G FNB needles, respectively. It was impossible to statistically compare the accuracy due to technical failure of the FNA group as the number of cases analyzed differed between them (19 vs. 20). The agreement between needles was 0.699, indicating good reliability (Table 4).

**TABLE 4:** Diagnostic efficiency of the 20-gauge FNB needle and the 22-gauge FNA needle

Needle	Sensitivity	Specificity	PPV	NPV	Accuracy	Kappa
22G FNA	77.8	100.0	100.0	20.0	78.9	0.689
20G FNB	88.9	100.0	100.0	50.0	90.0	

FNA=fine needle aspiration; FNB=fine needle biopsy; NPV=negative predictive value; PPV=positive predictive value

Two self-limited bleeds that did not require intervention were reported after the end of the last puncture; therefore, it was impossible to determine which needle caused the bleeding. No late or serious AEs occurred.

Of the 18 positive cases of malignancy, 10 died of pancreatic disease, three underwent palliative chemotherapy, one to neoadjuvant chemotherapy (imatinib for gastrointestinal stromal tumor), and two stopped follow-up after the detection of metastatic disease. There were also two cases of grade 1 pancreatic neuroendocrine tumors: one patient underwent surgical resection confirming G1 pNET and the other was being clinically followed up.

## DISCUSSION

This is the first controlled study to compare the new ProCore 20G FNB with the conventional 22G FNA fine needle for the diagnosis of pancreatic solid tumors. Previous studies compared the old ProCore 20G reverse-bevel FNB with the thinnest needle (25G FNA) available^33^ or reported data from uncontrolled studies^2^ ^,^ ^6^ ^,^ ^15^.

A recent study showed that the ProCore double forward-bevel needle has better performance than the previous generation^6^. Therefore, this new ProCore 20G device will soon replace the first-generation devices, which supports the assessment of its ability to obtain histological samples and achieve diagnostic accuracy.

The comparison between the use of the slow traction stylet and suction using the ProCore 20G needle showed that the two techniques have equivalent blood contamination and diagnostic accuracy^11^. A retrospective multicenter study showed that this needle achieved an 88% correct histological diagnosis rate for 50 subepithelial lesions^4^. The 85% sensitivity was greater than that of standard FNA^12^. This comparison of the 22G FNB needle (Acquire^®^, Boston Scientific) and the ProCore 20G showed similar diagnostic results except for the greater mean length of the histological nucleus per needle pass, which favored the 22G^22^. Those reports have all currently available data regarding the performance of the ProCore 20G needle; therefore, the present study retrospectively contributed to the overall findings and provides new results with greater evidence.

It was technically possible to perform EUS-guided puncture in all cases in the present study, in contrast to the 10% rate of technical failure due to the rigidity of the 20G needle that was reported in a recent work^22^. The only technical failure occurred with the 22G FNA needle and was detected during the microscopic examination. Therefore, there were no differences in the present study between needles regarding puncture difficulty, and the technical failure outcome may be associated with other factors such as lesion stiffness and location rather than the device used for the puncture. 

In addition to these difficulties, the endoscopist also subjectively classified the tissue amount. The 20G FNB provided significantly more samples classified macroscopically as suitable material. Although this can result from the caliber difference, it can also indicate that the FNB has an increased capacity to collect larger tissue samples. The histological assessment confirmed the latter hypothesis from two different perspectives: the mean sample size was significantly larger and the FNB provided more adequate samples than the 22G FNA needle.

On the other hand, the histological findings showed no differences in sensitivity. There are two hypotheses to explain this fact. First, the pathologist (MOB) is experienced and familiar with examining pancreatic lesions, which possibly improves the sensitivity of the puncture with the 22G FNA needle, thus reducing the difference between the needles. However, this result can change from favoring the ProCore 20G FNB in ​​centers with less experienced pathologists or those with general training. Additionally, this study had a small sample, which may have impaired our ability to identify an even greater difference.

Although it was impossible to show a statistical difference, other results, such as tissue adequacy, cellularity scores, and mean length of histological nucleus, were better with the ProCore 20G FNB and provided the pathologist with general training with suitable and representative histological material in addition to supporting additional molecular studies.

This assessment was reinforced in a recent study that collected samples using the FNB. In addition to greater diagnostic precision, there was greater agreement in the diagnosis of malignancy between academic and non-academic pathologists^32^. This information indirectly suggests that the ProCore 20G FNB provides greater confidence in the diagnosis of malignancy, especially for pathologists with general training, but this should be further studied.

It was difficult to separate the histological nucleus in samples collected with the ProCore 20G FNB needle, which can be associated with hypothetical failure resulting from intervening vessel injury. However, in the present study, there were no significant differences in bleeding of the samples collected by the two studied needles.

Regarding AEs, 2 cases of bleeding (10%) were reported, a rate that is higher than the mean reported rate of 0-2.9%^6^ ^,^ ^11^ ^,^ ^15^, and could indeed be associated with the characteristics of the ProCore 20G FNB in addition to the four punctures of each patient. Although the rate was high, the bleeding was self-limited and did not require any additional aggressive intervention.

Due to the technical failure of the 22G FNA, it was impossible to perform a statistical analysis to compare the accuracies of the needles, but the results of this study agree with those in the literature, which showed a better performance of the new ProCore 20G compared to the standard needle used in uncontrolled assessments^3^. Moreover, comparison of the 19G FNA needle with several other FNB needle models with the same caliber for the diagnosis of solid injuries showed an overall accuracy of 79% vs. 90% (p=0.039) for the 19G FNA and 19G FNB needles, respectively^20^. These results are like those reported in the present study, which suggests that a significant difference is possible in numerically comparable samples.

## CONCLUSION

The new ProCore 20G FNB double forward-bevel needle provided more adequate tissue samples with better cellularity scores and larger mean histological nucleus size without increasing puncture difficulty and bleeding episodes compared to the 22G FNA needle. Although there were no differences in diagnostic accuracy between the devices, the characteristics of the samples collected with the ProCore 20G FNB can favor centers with less experienced pathologists.
